# Minimum flow decomposition guided by saturating subflows

**DOI:** 10.64898/2025.12.11.693570

**Published:** 2026-01-22

**Authors:** Ke Chen, Abhishek Talesra, Sanchal Thakkar, Mingfu Shao

## Abstract

The minimum flow decomposition problem abstracts a set of key tasks in bioinformatics, including metagenome and transcriptome assembly. These tasks, collectively known as multi-assembly, aim to reconstruct multiple genomic sequences from reads obtained from mixed samples. The reads are first organized into a directed graph (e.g., overlap graph, splice graph), where each edge has an integer weight representing the number of supporting reads. By viewing the graph as a flow network, the underlying sequences and their abundances can be extracted through decomposition into a minimum number of weighted paths. Although this problem is NP-hard, prior work has proposed an efficient heuristic that transforms the graph by identifying nontrivial equations in the flow values. However, for graphs with complex structures, many equations cannot be fully resolved by existing mechanisms, leading to suboptimal decompositions. In this study, we revisit the theoretical framework of the flow decomposition problem and extend the equation-resolving mechanisms to jointly model all equations in the graph, enabling safe merge operations that iteratively simplify the graph. Experimental results demonstrate that our new algorithm substantially improves decomposition quality over existing heuristics, achieving near-optimal solutions for complex graphs, while running several orders of magnitude faster than the ILP formulation. Source code of our algorithm is available at https://github.com/Shao-Group/catfish-LP.git

## Full Text Availability

The license terms selected by the author(s) for this preprint version do not permit archiving in PMC. The full text is available from the preprint server.

